# Challenging management of craniocervical necrotizing fasciitis and descending mediastinitis in a low-resource setting: a case report and literature review

**DOI:** 10.1097/RC9.0000000000000231

**Published:** 2026-02-10

**Authors:** Selam Wubeshet Dessie, Elezer Berhanu Zewde, Tinsae Yidnekachew Tamiru, Dagem Habtamu Gessesse, Yabetse Girma Tessema, Leul Shigut Kejela

**Affiliations:** a Hayat Medical College, Addis Ababa, Ethiopia; b School of Medicine, College of Health Sciences, Addis Ababa University, Addis Ababa, Ethiopia; c Department of Surgery, Negele Arsi General Hospital and Medical College, Negele Arsi, Ethiopia

**Keywords:** cardiothoracic surgery, case report, craniocervical necrotizing fasciitis, descending mediastinitis, sepsis

## Abstract

**Introduction and importance:**

Craniocervical necrotizing fasciitis (CCNF) is a rare and rapidly progressive infection of the fascia and subcutaneous tissue in the head and neck. Clinical presentations range from fever, neck pain, and neck swelling to severe complications, such as descending necrotizing mediastinitis (DNM).

**Case presentation:**

A 40-year-old patient from rural Ethiopia presented with a 14-day history of neck pain, swelling, and fever. Physical examinations revealed fever, tachycardia, and tachypnea. After a CT scan confirmed CCNF–DNM, the patient underwent a series of surgical interventions and was started on broad-spectrum antibiotics, which led to the improvement of the patient’s condition over the subsequent week. However, limitations in providing a robust hemodynamic support, due to a lack of blood products in the facility, led to the patient’s unfortunate death, likely secondary to severe anemia and hypovolemic shock.

**Clinical discussion:**

CCNF–DNM is a rare and severe infection of the head and neck characterized by extensive tissue necrosis and rapid progression. Management is usually aggressive, involving broad-spectrum antimicrobial coverage, rapid surgical procedures, and intensive supportive care. Even with the combined efforts of a multidisciplinary team, CCNF–DNM carries a poor prognosis with a potential for catastrophic outcomes, especially in resource-limited settings.

**Conclusion:**

This report highlights the aggressive nature of CCNF–DNM and the importance of early recognition and intervention. Even with prompt surgery and antibiotics, survival is compromised without adequate supportive resources. Increased awareness, early referral to higher-level centers, and improved access to blood products and critical care are crucial for enhancing outcomes in low-resource settings.

## Introduction

CCNF is a severe and rapidly progressive soft tissue infection affecting the fascia and subcutaneous tissues of the head and neck, characterized by widespread fascial necrosis and systemic toxicity^[^1,2^]^. CCNF can present with various symptoms, including fever, neck pain, local erythema, and swelling, especially in the early stages^[^3,4^]^. As it advances, it may cause airway obstruction, sepsis, and spread to the mediastinum, leading to descending mediastinitis^[^5^]^. It is important to note that the clinical signs and symptoms of CCNF with mediastinal extension are nonspecific and may mimic deep neck infections or cellulitis. Therefore, a high index of clinical suspicion, along with supportive laboratory and imaging studies, is essential for early diagnosis and intervention.

HIGHLIGHTS

- Craniocervical necrotizing fasciitis (CCNF) is a rare and rapidly progressive infection of the fascia and subcutaneous tissue in the head and neck.
- CCNF can spread to the mediastinum and cause descending necrotizing mediastinitis (DNM)
- Management of CCNF–DNM is aggressive, involving broad-spectrum antibiotics and surgical debridement.
- Suprasternal transcervical mediastinal drainage and continuous irrigation may successfully be used when thoracotomy/VATS are unavailable
- Severe resource limitations can significantly impact outcomes of CCNF with DNM.

We report a case of a 40-year-old male from rural Ethiopia with 14 days of neck pain, swelling, and fever, diagnosed with CCNF–DNM after a thorough evaluation, with imaging evidence of an odontogenic source. This paper includes a comprehensive review of the literature on CCNF with DNM and highlights specific challenges in managing these cases, particularly in severely resource-limited settings, where access to microbiology, advanced surgical techniques, and blood products is often restricted. Furthermore, this report describes the use of suprasternal transcervical mediastinal drainage and continuous irrigation as a potential alternative and innovative approach when thoracotomy or video-assisted thoracoscopic surgery (VATS) are unavailable. This paper has been reported in line with the SCARE criteria^[^6^]^

## Case presentation

A healthy 40-year-old male from rural Ethiopia was referred to our hospital with 14 days of neck swelling, pain, and fever. A week prior, he was treated at a local hospital with an unspecified surgical procedure (likely incision and drainage) and antibiotics, but his condition worsened, prompting referral. On arrival, he was tachycardic (130–140 bpm), tachypneic (26 breaths/min), and had a 38°C fever, corresponding to a systemic inflammatory response syndrome (SIRS) score of 3.

**Figure 1.**
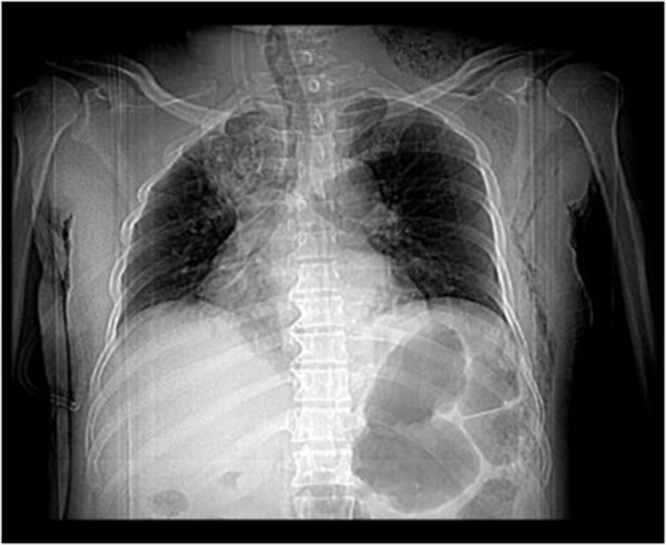
Preliminary chest X-ray showing a right superior mediastinal opacity with multiple left chest wall gas shadows.

**Figure 2.**
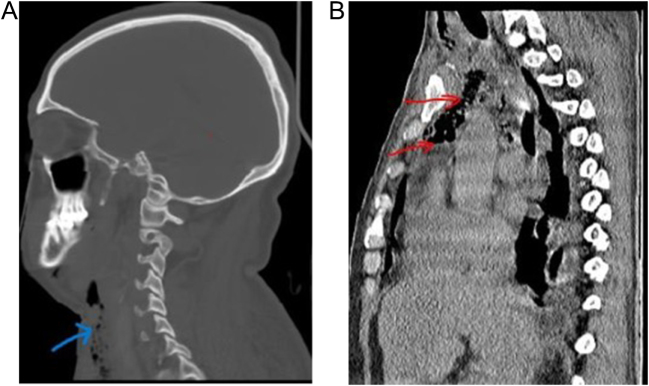
(A and B). CT scan sagittal view showing the extensive pretracheal subcutaneous tissue gas collection (A) extending downward into the superior mediastinum (B).

**Figure 3.**
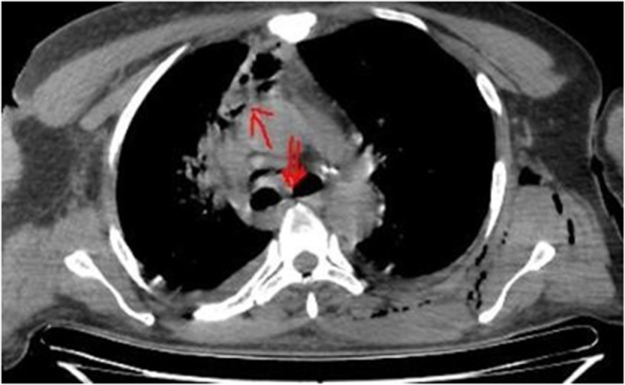
Axial mediastinal window with descending necrotizing mediastinitis (single red arrow) extending to the level of the carina (double red arrow).

**Figure 4.**
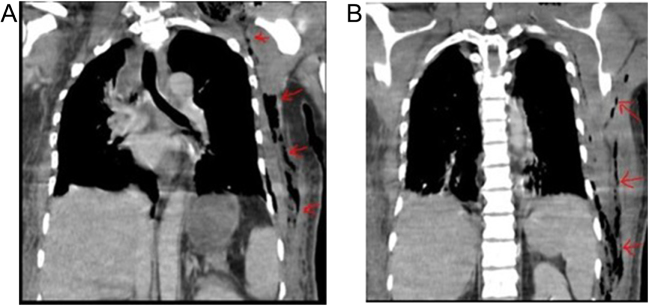
(A and B) Coronal chest CT image shows the lateral chest wall and suprascapular extension of the subcutaneous emphysema.

**Figure 5.**
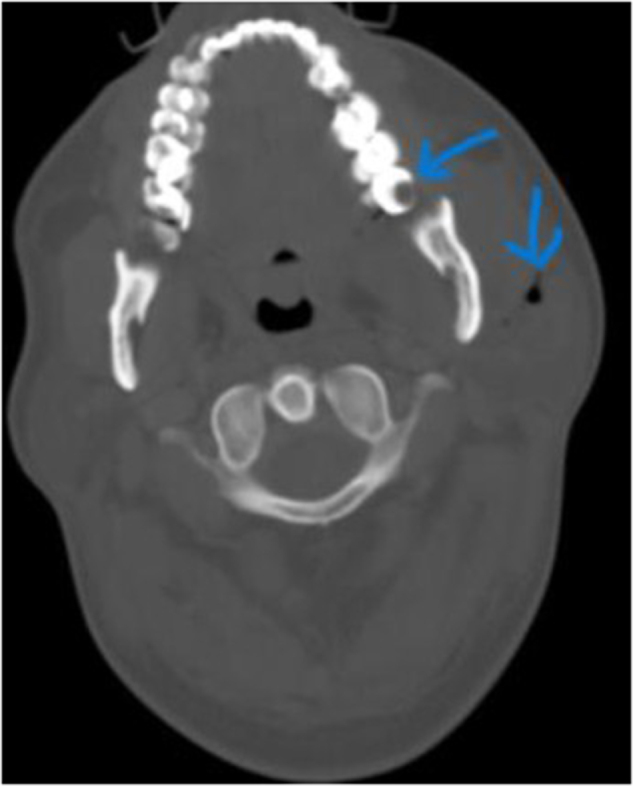
Axial bone CT scan image showing a last molar dental cavitation and periodontal abscess.

**Figure 6:**
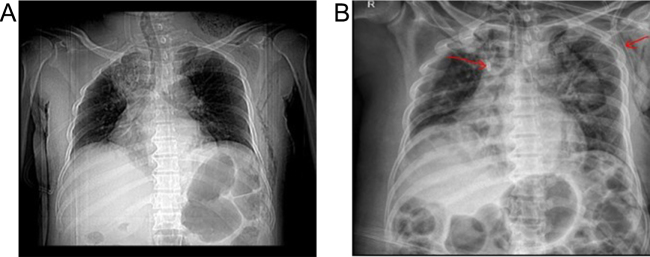
(A) Initial chest X-ray showing a right superior mediastinal opacity. (B) Post-tracheostomy and mediastinal irrigation tube placement showing resolution of right superior mediastinal opacity. Red arrow showing the drainage tube at the superior mediastinum and another in the axillary fold.

Physical examination revealed significant soft tissue swelling with blanching that extended from the neck and left lateral chest down to the mid-thigh. Additionally, a 2 cm surgical incision over the left submandibular area and a 4 cm incision over the posterolateral side of the left 6th rib, both with foul-smelling discharge, were also noted. Chest auscultation revealed bilateral reduced air entry in the lower lung fields, but no subcutaneous emphysema was noted. Laboratory investigations showed a white blood cell (WBC) of 5500/μL (84.9% neutrophils), hemoglobin (Hgb) of 11 g/dL, a hematocrit of 31%, and an elevated CRP (15 mg/dL). Other laboratory tests, including renal function, electrolytes, and viral markers, were normal, but a blood culture was unavailable. Chest X-ray showed a right superior mediastinal opacity and gas shadows on the left chest wall (Fig. 1 and Fig. 6A). CT scans revealed extensive neck, chest wall, mediastinal subcutaneous emphysema, bilateral pyothorax, and chest wall abscesses (Figs. 2–4). Axial CT scan images also demonstrated last molar dental cavitation with an associated periodontal abscess, suggesting an odontogenic source of infection (Fig. 5).

Following a diagnosis of Type I CCNF-DNM, the patient underwent five major surgeries. The first surgery lasted for 150 minutes and consisted of four major procedures involving bilateral neck exploration, tracheostomy, mediastinal washout, and bilateral tube thoracostomy. The patient was started on broad-spectrum antimicrobial therapy with vancomycin 1 gm IV twice daily, meropenem 2 gm IV three times daily, and clindamycin 600 mg IV three times daily, along with omeprazole 40 mg IV daily and unfractionated heparin 7500 IU SC twice daily for gastrointestinal and deep vein thrombosis prophylaxis.

The first procedure aimed at neck exploration and incision to drain the primary focus. A low collar incision and bilateral 4 cm incisions along the anterior border of the sternocleidomastoid muscle were made, revealing extensive necrosis of the deep cervical fascia and strap muscles. The submandibular incision was extended parallel to the mandible edge to open the submandibular and parapharyngeal spaces. The collar and submandibular incisions were connected using a Penrose drain, and thorough irrigation cleansed all necrotic tissues and debris, with antiseptic solutions also applied.

The second procedure focused on the mediastinum and pleural cavity. After considering a right thoracotomy versus a low collar approach to access the mediastinum, the latter was favored due to the absence of blood products and the reduced morbidity. Mediastinal drainage was performed through suprasternal access, with an irrigation tube placed in the mediastinum for continuous flushing of necrotic contents outside the operating room (Fig. 6). Cleaning with peroxide and iodine solutions was performed. Bilateral tube thoracostomy was also carried out to drain the pleural space.

The third procedure addressed the airway, where strap muscles and the thyroid isthmus were divided. The second and third tracheal rings were palpated, and a Bjork flap was created in the standard fashion. The endotracheal tube was removed under direct visualization, and a No. 7 tracheostomy tube was inserted and connected to the anesthesia machine.

The fourth procedure aimed at source control and involved serial limiting incisions. The incisions were made across the left shoulder, chest wall, lateral abdominal wall, and extended down to the left mid-thigh, revealing dishwater-like fluid. Despite the significant intraoperative bleeding, the patient tolerated the surgery well and was transferred to the ICU in stable condition. Due to the unavailability of blood products at the time, the patient was given intravenous fluid.

The subsequent surgeries involved repeated debridement and washout, performed 48 hours apart. Throughout these procedures, his condition improved significantly. By the fifth operation, mediastinal discharge had ceased, and incisions at the submandibular and collar regions appeared clean with ample granulation tissue. Incisions at the sternal notch, thigh, hip, and left arm were also clean and contained viable tissue. The left flank incision showed improvement but still had limited discharge. However, debris and pus from the shoulder blade and joint persisted, requiring extensive debridement, including the anterior shoulder joint. Careful wound cleaning with antiseptics and redebridement of the neck and mediastinum were performed, with drains placed to facilitate irrigation. Incisions across the left thigh, left arm, and sternum were eventually closed in a delayed primary closure.

The patient remained in the ICU, receiving supportive care including antibiotics, omeprazole, and heparin for prophylaxis. He also had regular wound and tracheostomy care. During his stay in the ICU, he was transfused with five units of cross-matched blood to address anemia caused by the multiple trips to the operating theatre. Hemoglobin levels showed a progressive decline despite few transfusions. Immediate postoperative hemoglobin was 7.8 g/dL (hematocrit 20.3%), on day 7 it was 6.7 g/dL (hematocrit 20.1%), and on day 15 it decreased further to 4.0 g/dL.

By the second week, he showed clinical improvement, evidenced by normalization of vital signs (including the resolution of fever), restored appetite, and resolution of infection around the limiting incisions, allowing for delayed primary closure and weaning off of the ventilator on day 13. However, on day 15, he developed hemodynamic instability with tachycardia, severe hypotension, oxygen desaturation, and his hemoglobin dropped to 4 g/dL. Unfortunately, the patient ultimately died from severe anemia and hypovolemic shock, in the absence of sufficient blood products for successful stabilization efforts.

## Discussion

CCNF is a severe, rapidly progressive bacterial infection of the fascia in the head and neck^[^1,2^]^. It is characterized by widespread fascial necrosis and extreme systemic toxicity. CCNF is commonly complicated by DNM, potentially leading to airway compromise, sepsis, and multiorgan failure. Although only 1–10% of necrotizing fasciitis cases occur in the craniofacial region, 40–45% of patients end up developing mediastinal extensions^[^7^]^.

CCNF typically originates from an odontogenic or oropharyngeal source, then spreads along cervical fascial planes into the mediastinum^[^8^]^. The infection is usually polymicrobial, involving both aerobes and anerobes of the oral flora such as streptococci, staphylococci, Enterobacterales, Bacteroides, Fusobacterium, and Peptostreptococcus^[^8,9^]^. Bacterial toxins and gas production play a crucial role in rapid tissue necrosis. In our patient, CT imaging demonstrated a last molar dental cavitation with periodontal abscess, supporting an odontogenic origin of infection (Fig. 5). The anatomical continuity of cervical fascial spaces into the mediastinum facilitates its spread, leading to DNM. The term “descending necrotizing mediastinitis” was first used by Pearse in 1938^[^10,11^]^. Its diagnostic criteria were proposed by Estrera *et al* in 1983^[^11,12^]^: (I) clinical manifestation of severe infection; (II) radiological abnormalities characteristic of the disease; (III) surgical or postmortem evidence of DNM; and (IV) correlation between oropharyngeal infection and the development of DNM.

**Figure 7.**
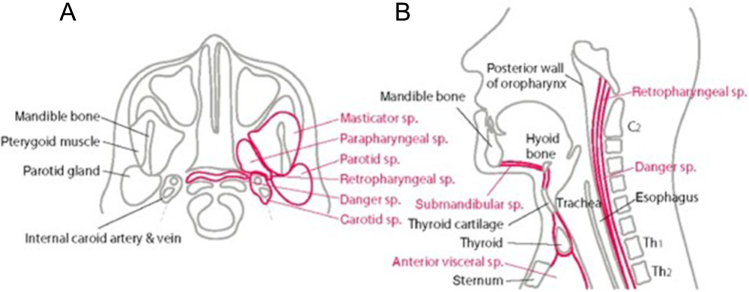
Anatomic pathways of infection from neck spaces into the mediastinum. The parapharyngeal space (center) communicates with the pretracheal (anterior), carotid (lateral), and retropharyngeal (posterior) spaces. Infection in the retropharyngeal/danger space can descend to the posterior mediastinum. ©Japanese Association for Acute Medicine^[^8^]^.

DNM is classified based on the extent of mediastinal spread. Endo *et al* described type I (localized DNM) as an infection confined to the upper mediastinum above the carina, and type II (diffuse DNM) as involving the lower mediastinum. Type II is further subdivided into IIA (involving the anterior compartment) and IIB (affecting both anterior and posterior compartments)^[^5,13^]^. As illustrated in Fig. 7, there are three principal routes of mediastinal extension: the pretracheal route to the anterior mediastinum, the lateral pharyngeal route to the middle mediastinum, and the retropharyngeal/danger space route to the posterior mediastinum (Fig. 7). Under the influence of negative intrathoracic pressure, the infection can track downward swiftly as there are no anatomical barriers to the thorax^[^8^]^. In our case, imaging findings were consistent with type 1 DNM, which helped guide the decision for a suprasternal transcervical approach rather than a thoracotomy. Due to this anatomy, early cases of CCNF may give deceptive minimal skin changes despite deep tissue necrosis.

Diagnosing CCNF with mediastinal extension at presentation is notoriously difficult. Early symptoms such as fever, neck pain, and swelling are often nonspecific and mimic ordinary deep neck infections or cellulitis^[^3,4^]^. Skin necrosis or crepitus usually appear late, so a high index of clinical suspicion is required. Studies show that only 15–34% of patients with CCNF and DNM are correctly diagnosed upon admission^[^8^]^. Laboratory findings usually include marked leukocytosis, neutrophilia, and elevated inflammatory markers. The Laboratory Risk Indicator for Necrotizing Fasciitis (LRINEC) score ≥6, as described in Table 1, is recommended to prompt evaluation for NF and can help support early diagnosis^[^14^]^ (Table1). However, recent literature indicates that the LRINEC score’s diagnostic accuracy is inconsistent. A meta-analysis by Fernando *et al* (2019), pooling 23 studies, reported a sensitivity of approximately 68% and a specificity of about 85% for LRINEC ≥6, concluding that the score has poor sensitivity and should not be used alone to rule out NSTI^[^15^]^. A narrative review similarly noted variable sensitivity ranging from 43% to 80%, raising concerns especially in immunocompromised patients^[^16^]^. Overall, studies suggest that high LRINEC scores can raise suspicion and correlate with severity, but low scores do not reliably exclude necrotizing fasciitis^[^15,17^]^. Similarly, in our patient, despite a low initial LRINEC score of 5 and a normal white blood cell count (5500/µL), the presence of systemic toxicity and discharge from previous surgical sites, and a SIRS score of 3 raised strong clinical suspicion, which was subsequently confirmed by a CT scan.

**Table 1:** Laboratory risk indicator for necrotizing fasciitis score^[^14^]^

Variable		Score
C- reactive protein (mg/L)	<150	0
	≥150	4
Total white blood cell count (/mm^3^)	<15	0
	15-25	1
	>25	2
Hemoglobin (g/dL)	13.5	0
	11-13.5	1
	<11	2
Sodium (mmol/L)	≥135	0
	<135	2
Creatinine (μmol/L)	≤141	0
	>141	2
Glucose (mmol/L)	≤10	0
	>10	1

A CT scan is the imaging modality of choice once NF is suspected. A contrast-enhanced neck and chest CT can reveal fascial thickening, fluid collections, gas in tissues, and continuity of infection into the mediastinum^[^8^]^. MRI has high sensitivity, but is impractical in an unstable patient. In our case, CT imaging was critical not only for confirming the diagnosis, but also for identifying the extent of mediastinal involvement and revealing an odontogenic source, which directly influenced both surgical planning and antibiotic selection. Given the poor early sensitivity of clinical exam, we agree with experts that any deep neck infection with disproportionate pain, rapid swelling, or systemic signs should prompt consideration of NF and CT imaging^[^4,8^]^.

Management of CCNF with DNM is usually aggressive and involves multidisciplinary teams. The focus of management should be on rapid surgical source control and broad-spectrum antimicrobial coverage, along with intensive supportive care. Multiple, rapid serial debridements of all necrotic tissue may be required to ensure no potential source of infection is left behind^[^18,19^]^. This was evident in our patient as well, who underwent repeated surgical explorations and debridements. Studies indicate that a special focus on addressing mediastinal involvement is needed to improve treatment outcomes^[^18–20^]^. Sandner *et al* reported thoracotomy in four of six DNM cases, combined with cervical drainage^[^19^]^. Other series emphasize transcervical with subxiphoid or VATS approaches, depending on the extent of the infection^[^8,20^]^. Recent data, such as Yun *et al*, suggest that VATS mediastinal drainage can achieve outcomes comparable to open thoracotomy, with shorter durations of antibiotics, drainage, and hospital stays^[^20^]^. In our patient, thoracotomy was considered as a possible approach to address mediastinal involvement. However, due to limited resources and a lack of sufficient blood products, thoracotomy was not feasible. Additionally, VATS was not an option because of the absence of trained physicians and the necessary equipment. Given these constraints and that the patient had localized DNM (Type 1), we opted for drainage through suprasternal transcervical approach, along with an irrigation tube placement for continuous flushing of necrotic contents outside of the operating room. Although unconventional, this resource-adapted approach led to radiological improvement with the resolution of the right superior mediastinal opacity seen in the initial chest X-ray (Fig. 6). Further studies are needed to evaluate this method as a treatment option for Type 1 DNM, particularly in resource-limited settings like ours, where thoracotomy and VATS may not be available.

Antibiotic therapy must cover polymicrobial and potentially resistant pathogens. Empirical regimens for necrotizing soft tissue infections typically include a carbapenem or piperacillin-tazobactam for broad Gram-negative and anaerobic coverage, plus an agent active against methicillin-resistant *Staphylococcus aureus* (MRSA)^[^21^]^. Clindamycin is added for its antitoxin effect, especially if Group A strep or clostridia are involved^[^21,22^]^. A review of published cases of DNM found that carbapenems and clindamycin were the most commonly used agents^[^8^]^. Our choice of vancomycin 1 gm IV BID (for MRSA), meropenem 2 gm IV TID, and clindamycin 600 mg IV TID also aligns with these literature recommendations. Generally, prolonged courses of antibiotics are needed due to the severity of the infection, with an average of 27 days reported in a retrospective analysis of 25 patients with DNM treated at a single center^[^20^]^.

We cannot emphasize enough the importance of supportive ICU care. Patients often require intubation or tracheostomy due to airway compromise from surrounding edema and soft-tissue swelling. Studies have revealed that a decreasing perioperative hemoglobin level in patients with necrotizing fasciitis, particularly <6 g/dL, is associated with a higher mortality rate^[^23^]^. In our patient, hemoglobin progressively declined from 7.8 g/dL postoperatively to 6.7 g/dL on day 7 and ultimately to 4.0 g/dL on day 15, despite transfusions. Therefore, aggressive fluid resuscitation, transfusions with blood products, vasopressors, and ventilatory support should be provided as needed. Nutritional support is also essential in these hypercatabolic patients. Lastly, frequent wound care, including open packing and negative-pressure dressings, is necessary after debridement. Successful management depends on early recognition, broad-spectrum antibiotics, serial surgical debridements, and coordinated supportive care in the ICU^[^4,22^]^.

Despite aggressive management strategies, overall mortality remains high. In the initial report where cervical necrotizing fasciitis with DNM was first described, the mortality was nearly 49%^[^10^]^. Recent literature also reports a high mortality rate; for example, de Leyva *et al* reported that cervical necrotizing fasciitis mortality can reach up to 41% when associated with DNM and as high as 64% when complicated by sepsis^[^4^]^. However, some more recent series showed improvement in mortality. Yun *et al* reported only 12% in-hospital mortality (3 of 25) in a cohort undergoing aggressive surgery and ICU care, while Sumi’s Japanese review found 5.6% overall mortality^[^8,20^]^. Table 2 summarizes key recent series reporting mortality rates with the surgical management approaches used (Table 2).

**Table 2:** Reported series of cervical necrotizing fasciitis with mediastinal spread (DNM); surgical approaches

Study (year)	*N* (DNM cases)	Surgical management	Mortality (%)
Al-Ali *et al* (2018)^[^18^]^	6 (4 DNM)	Repeated neck debridements; mediastinal washout if needed; antibiotics	16.7% (1/6)
Sandner *et al* (2006)^[^19^]^	6 (6 DNM)	Cervical drainage + thoracotomy in 4 cases; tracheostomy in 4	33% (2/6)
Sumi (2014)^[^8^]^	51 (DNM)	Various: transcervical (25 patients), VATS (21), subxiphoid catheters (15), thoracotomy (21)	5.6% (3/54)
Yun *et al* (2023)^[^20^]^	25 (25 DNM)	Transcervical drainage (15); VATS (10)	12% (3/25)

Unfortunately, our patient’s course was fatal, likely due to severe anemia and hypovolemic shock. We believe that the combination of blood loss from repeated debridements and systemic capillary leak caused the critical anemia observed in our patient. If not treated, this can lead to shock, even if the infection is improving, as seen in our case. Additionally, the limited blood bank resources at our center posed a significant challenge in providing adequate resuscitation. Moreover, the initial surgical procedure, which seemed to have been an attempt to drain through a small incision at the local hospital, was insufficient to control the progression of the infection. This case highlights that, even when appropriate surgical and antimicrobial management is instituted, systemic resource limitations, particularly the lack of blood products for transfusions, can be the decisive factor in patient survival.

## Conclusion

Our case of a 40-year-old Ethiopian patient with CCNF advancing to DNM aims to highlight several key issues. It emphasizes the aggressive nature of the condition and the importance of maintaining a high index of clinical suspicion for early diagnosis. Relying on scoring systems like LRINEC may assist with our suspicion but should not be used exclusively to rule out necrotizing soft tissue infections. This case also demonstrates that managing these patients requires aggressive source control and appropriate broad-spectrum antibiotics coverage. In addition, we describe the use of a suprasternal transcervical approach with continuous mediastinal irrigation as a potential alternative when thoracotomy or VATS are not feasible in resource limited environments. Finally, the tragic outcome of our patient illustrates that adequate hemodynamic support, including timely blood transfusions, must be a crucial component of management plans. This report underscores that, even when timely surgical and antimicrobial treatment is provided, systemic resource limitations can critically influence outcomes. Increased awareness, early referral to higher-level centers when possible, and improved access to blood products and critical care resources are essential to improving survival in CCNF-DNM in low-resource settings.

## Data Availability

Not applicable. This manuscript does not report data generation or analysis.
